# Toxic Potential of Synthesized Graphene Zinc Oxide Nanocomposite in the Third Instar Larvae of Transgenic *Drosophila melanogaster (hsp70-lacZ)Bg* ^*9*^

**DOI:** 10.1155/2014/382124

**Published:** 2014-06-15

**Authors:** Yasir Hasan Siddique, Wasi Khan, Saba Khanam, Smita Jyoti, Falaq Naz, Rahul, Braj Raj Singh, Alim H. Naqvi

**Affiliations:** ^1^Drosophila Transgenic Laboratory, Section of Genetics, Department of Zoology, Faculty of Life Sciences, Aligarh Muslim University, Aligarh, Uttar Pradesh 202002, India; ^2^Centre of Excellence in Materials Sciences (Nanomaterials), Department of Applied Physics, Z.H. College of Engineering & Technology, Aligarh Muslim University, Aligarh, Uttar Pradesh 202002, India

## Abstract

In the present study the graphene zinc oxide nanocomposite (GZNC) was synthesized, characterized, and evaluated for its toxic potential on third instar larvae of transgenic *Drosophila melanogaster (hsp70-lacZ)Bg* ^*9*^. The synthesized GZNC was characterized by X-ray diffraction (XRD), Fourier transform infrared spectroscopy (FTIR), thermogravimetric analysis (TGA), scanning electron microscopy (SEM), and transmission electron microscopy (TEM). The GZNC in 0.1% dimethyl sulphoxide (DMSO) was sonicated for 10 minutes and the final concentrations 0.033, 0.099, 0.199, and 3.996 *μ*g/*μ*L of diet were established. The third instar larvae were allowed to feed on it separately for 24 and 48 hr. The *hsp70* expression was measured by o-nitrophenyl-*β*-D-galactopyranoside assay, tissue damage was measured by trypan blue exclusion test, and *β*-galactosidase activity was monitored by *in situ* histochemical *β*-galactosidase staining. Oxidative stress was monitored by performing lipid peroxidation assay and total protein estimation. Ethidium bromide/acridine orange staining was performed on midgut cells for apoptotic index and the comet assay was performed for the DNA damage. The results of the present study showed that the exposure of 0.199 and 3.996 *μ*g/*μ*L of GZNC was toxic for both 24 hr and 48 hr of exposure. The doses of 0.033 *μ*g/*μ*L and 0.099 of GZNC showed no toxic effects on its exposure to the third instar larvae for 24 hr as well as 48 hr of duration.

## 1. Introduction

Nanoparticles (NPs) have novel properties, large specific surface, and high reaction activity [1, 2]. Due to the rapid development of nanotechnology, nanomaterials with various shapes and diameters have been prepared for the use in some industrial products and commodities [3]. Besides having applications in drug delivery, cell imaging, and cancer therapy, metal oxide nanoparticles have been manufactured for both industrial and household applications [3]. It has been reported that zinc oxide nanoparticles have negative impacts on the survival and growth of organisms [4]. The physical parameters of nanoparticles can affect their nonspecific uptake in cells, with potential to induce cellular responses [5]. Graphene is an allotrope of carbon and is being used in nanocomposites due to its intrinsic properties [6]. It is typically free of impurities as compared to carbon nanotubes, having an advantage of being used in the construction of reliable sensors as well as storage devices [6, 7]. Due to the wide range of potential applications and novel properties, graphitic nanomaterials such as carbon nanotubes, fullerenes, and more recently graphene have gained a great deal of interest in the scientific community [8]. The biological applications and potential adverse effects of graphene still remain to be clear and need a detailed study that would provide information in this regard. However, till date the reports on the toxic evaluation of its nanocomposites are warranted. In the present study the graphene zinc oxide nanocomposite (GZNC) was synthesized, characterized, and evaluated for its toxic potential on third instar larvae of transgenic* Drosophila melanogaster (hsp70-lacZ)Bg* ^*9*^ as a model.

## 2. Materials and Methods

### 2.1. Synthesis of Graphene Zinc Oxide Nanocomposites

Graphene oxide (GO) was synthesized from 2 g of natural graphite powder by using a modified Hummer and Offeman's method [9]. The synthesized GO was centrifuged and washed successively with 4% HCl until it reached neutral pH value. The obtained GO was dried in vacuum oven at 70°C for overnight and used for the synthesis of GZNC. For the synthesis of GZNC, 1 g of GO was dissolved in 100 mL of MilliQ water using ultrasonicator. The zinc nitrate hexahydrate (25 mM) was added into the dissolved GO solution under vigorous stirring and pH was increased to 11 using NaOH solution. The reaction mixture was stirred for 1 hour at 150°C and obtained GZNC was centrifuged. The synthesized GZNC was washed several times with water and ethanol thoroughly. The synthesized GZNC was dried and stored for further study.

### 2.2. Characterizations of GZNC

The X-ray diffraction (XRD) pattern of powder sample of GZNC was recorded on MiniFlex II benchtop XRD system (Rigaku Corporation, Tokyo, Japan) operating at 30 kV and a current of 15 mA with Cu K*α* radiation (*λ* = 1.54 Å). The diffracted X-rays were recorded from 20° to 80° at 2*θ* angles. For the FTIR spectroscopic measurement of GZNC powder was mixed with spectroscopic grade potassium bromide (KBr) in the ratio of 1 : 100 and spectra were recorded in the range of 400–4000 wavenumber (cm^−1^) on Perkin Elmer FTIR Spectrum BX (PerkinElmer Life and Analytical Sciences, CT, USA) in the diffuse reflectance mode at a resolution of 4 cm^−1^ in KBr pellets. The synthesis of GZNC in ethanol solution was monitored by measuring the absorbance (A) using UV-visible spectrophotometer (Perkin Elmer Life and Analytical Sciences, CT, USA) in the wavelength range of 200–800 nm. The thermal stability of the GZNC was investigated by thermogravimetric analysis (TGA) at a heating rate of 10°C/min under nitrogen atmosphere. The microstructure and morphology analyses of sample were done using a JEOL transmission electron microscope (TEM) (JEM-2010) and scanning electron microscope (SEM) (JSM-6510LV) equipped with an energy dispersive spectrometer (EDS).

### 2.3. Fly Strain

A transgenic* Drosophila melanogaster (hsp70-lacZ)Bg* ^*9*^ line that expresses bacterial *β*-galactosidase as a response to stress was used in the present study [10]. The flies and larvae were cultured on standard* Drosophila* food containing agar, corn meal, sugar, and yeast at 24  ±  1°C [11, 12].

### 2.4. Experimental Design

GZNC in 0.1% DMSO was sonicated for 10 min and the final concentrations 0.033, 0.099, 0.199, and 3.996 *μ*g/*μ*L of diet were established. The larvae were allowed to feed on diet separately for 24 and 48 hr. Untreated and negative control (0.1% DMSO) were also run simultaneously.

### 2.5. Soluble o-Nitrophenyl-*β*-D-Galactopyranoside Assay

The expression of* hsp70 *provides a measurement of cytotoxicity [13, 14]. The method described by Nazir et al. [11] was used in this study. After washing in phosphate buffer, larvae were placed in a microcentrifuge tube (20 larvae/tube; five replicates/group), permeabilized for 10 min by acetone, and incubated overnight at 37°C in 600 *μ*L of ONPG staining buffer. Following incubation, the reaction was stopped by adding 300 *μ*L of Na_2_CO_3_. The extent of the reaction was quantified by measuring absorbance at 420 nm.

### 2.6. Trypan Blue Exclusion Test

The extent of tissue damage in larvae caused by the exposure to different concentrations of GZNC was assayed by a dye exclusion test [11, 15]. Briefly, the internal tissues of larvae were explanted in a drop of Pole's salt solution (PSS), washed in phosphate buffer saline (PBS), stained in trypan blue (0.2 mg/mL in PBS) for 30 min, washed thoroughly in PBS, and scored immediately for dark blue staining. About 50 larvae per treatment (10 larvae per dose; 5 replicates per group) were scored for the trypan blue staining on an average composite index per larvae: no color = 0; any blue = 1; darkly stained = 2; large patches of darkly stained cells = 3; or complete staining of most cells in the tissue = 4 [15].

### 2.7. *In Situ* Histochemical *β*-Galactosidase Activity

The larvae (10 larvae/treatment; 5 replicates/group) were dissected out in PSS and X-gal staining was performed using the method as described by Chowdhuri et al. [13]. The tissue explants were fixed in 2.5% glutaraldehyde, washed in 50 mM sodium phosphate buffer (pH 8.0), and stained overnight in X-gal staining solution at 37°C in the dark.

### 2.8. Preparation of Larval Homogenate for Lipid Peroxidation Assay and Total Protein Content

The larvae (10 larvae/experiment; 5 replicates/group) were homogenized in 1 mL of cold homogenizing buffer (0.1 M phosphate buffer containing 0.15 M KCl; pH 7.4). The supernatant after centrifugation at 9000 g was used for estimating lipid peroxidation and total protein content.

### 2.9. Lipid Peroxidation Assay

Lipid peroxidation assay was performed using 1,1,3,3-tetramethoxypropane as a standard according to the method described by Siddique et al. [16, 17].

### 2.10. Protein Estimation

Estimation of protein level in all the treated groups as well as control groups was done according to the method of Bradford [18], using bovine serum albumin (BSA) as a standard.

### 2.11. Assay to Detect Apoptosis

The apoptotic cells were analyzed by staining with an ethidium bromide (EB) and acridine orange (AO) staining according to the procedure described in our earlier published work [17]. About 100 cells were scored per treatment (5 replicates/group) for estimating the apoptotic index and expressed in percentages [19].

### 2.12. Analysis of DNA Damage by Comet Assay

The comet assay was performed according to Mukhopadhyay et al. [20]. The midguts from 20 larvae were explanted in PSS. PSS in microcentrifuge tube was replaced by 300 *μ*L of collagenase (0.5 mg/mL in PBS, pH 7.4) and kept for 15 min at 25°C. The cell suspension was prepared by washing three times in PBS and finally the cells were suspended in 80 *μ*L of PBS. The cell viability was checked by performing trypan blue assay before beginning the experiment and the assay was performed according to the procedure described in our earlier published work [17, 21]. Each experiment was performed in triplicate and the slides were prepared in duplicate. Twenty-five cells per slide were randomly captured at a constant depth of the gel, and mean tail length was calculated to measure DNA damage by using Comet Score 1.5 Software (Comet Score v1.5 Software, TriTek Corporation, Sumerduck).

### 2.13. Statistical Analysis

Student's *t*-test and regression analysis were performed by using commercial software Statistica from Stat-Soft Inc.

## 3. Results

### 3.1. X-Ray Diffraction (XRD)

**Figure 1:**
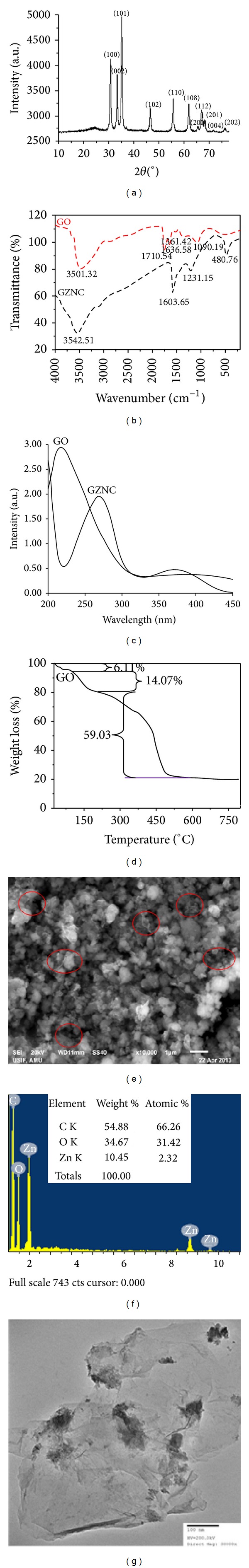
(a) XRD pattern of synthesized GZNC. (b) FTIR spectrum of GZNC. (c) UV-visible absorption spectra of GO and GZNC. (d) Thermogravimetric analysis spectrum shows the thermal behavior of the GZNC. (e) SEM image of GZNC. (f) EDS spectrum of GZNC. (g) TEM image of GZNC.

X-ray diffraction (XRD) measurement was employed to investigate the phase and structure of the synthesized GZNC sample. The XRD pattern of the GZNC is shown in Figure 1(a); there were eleven main diffraction peaks located at 2*θ* = 31.33°, 34.0°, 35.34°, 46.43°, 55.68°, 61.91°, 65.46°, 67.18°, 68.14°, 71.67°, and 76.36° which correspond to the crystal planes (100), (002), (101), (102), (110),  (103), (200), (112), (201), (004), and (202) of the hexagonal wurtzite structure of ZnO reported in JCDDS card (number 36–1451, *a* = 3.249 Å, and *c* = 5.206 Å), respectively. The XRD data of GZNC indicated the absence of any other impurities, which reflected its high quality. The average crystallite size (*D*) of ZnO nanoparticles was calculated using the Debye-Scherrer formula:

(1) $$\begin{array}{c} D\;=\frac{k\lambda }{\beta \cos\theta }, \end{array}$$

where *k* = 0.9 is the shape factor, *λ* is the X-ray wavelength of Cu K*α* radiation (1.54 Å), *θ* is the Bragg diffraction angle, and *β* is the full width at half maximum height (FWHM) of the (111) plane diffraction peak. The calculated average crystallite size was found to be ~9 nm. Further the main characteristic diffraction peak was observed in the synthesized GZNC at 35°, which corresponds to (101) reflection plane of graphene with basal spacing of *d*_022_ = 3.62 Å. The XRD data clearly indicates the successful synthesis of GZNC in this study.  Figure 1(b) shows the FTIR spectrum of synthesized GZNC. The absence of the characteristic peaks of carboxyl group at 1710 cm^−1^, hydroxide group at 1361 cm^−1^, and C–O (alkoxy) groups at 1090 cm^−1^ indicates that most oxygen-containing functional groups in the GO were removed. The spectrum of the GZNC shows an absorption band at 1603 cm^−1^, C=C stretching, indicating the restoration of the graphene network on reduction. The presence of the absorption band at 480 cm^−1^ in GZNC is identified as ZnO nanoparticles (NPs).

### 3.2. Optical Characteristics

The UV-visible spectra of GO and GZNC are shown in Figure 1(c). The GO sample showed the absorption peak at ~220 nm and a shoulder at ~280 nm. The peak at 220 nm is assigned to the pi to anti-pi (*π* → π*) transition of the aromatic C–C bonds and the shoulder at 278 nm is assigned to the *n* to anti-pi (*n* → π*) transitions of the C=O bonds. The UV spectrum of GZNC showed two distinct peaks at ~270 and ~370 nm which corresponds to the excitation of the *π*-plasmon of the graphitic structure and ZnO NPs characteristics, respectively. The graphene absorption peak is entirely different from GO peak and red shifted to 270 nm which indicates the fully reduced graphene from GO. The absorption data confirmed again the successful synthesis of GZNC in this study.

### 3.3. Thermal Characteristics

The TGA curve of GZNC is shown in Figure 1(d). The weight loss of 6.11% occurring at about 63°C is associated with adsorbed water. Pyrolysis of the labile oxygen-containing functional groups at about 200°C accounts for 14.07% of weight loss. The thermal decomposition observed in the temperature range 200–500°C with 59.03% of weight loss is attributed to the pyrolysis of the carbon skeleton. These results illustrate that GZNC has a remarkable thermal stability (Figure 1(d)).

Figure 1(e) shows the SEM image of GZNC. It can be seen that most of the graphene nanosheets are curled and entangled together. The results reveal that the presence of graphene sheet is uniformly distributed in the sample. Moreover ZnO NPs were also clearly shown in the low dimension and well dispersed all over the sample. The EDS spectrum demonstrates that ZnO contents are uniformly doped into the graphene matrix. An oxygen peak at about 0.52 keV, Zn signals at about 1 keV and 8.6 keV, and presence of the graphene (i.e., carbon) at 0.25 keV were observed in the EDS spectrum as displayed in Figure 1(f). These results were consistent with the analysis of the XRD data.

**Figure 2:**
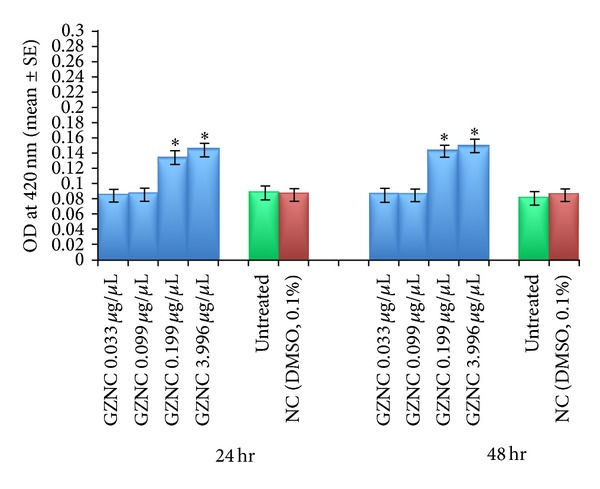
*β*-Galactosidase activity measured in transgenic* Drosophila melanogaster (hsp70-lacZ)Bg* ^*9*^ third instar larvae exposed to different doses of graphene zinc nanocomposite (GZNC) for 24 and 48 hr. *Significant at *P* < 0.05 with respect to untreated larvae (GZNC: graphene zinc nanocomposite; NC: negative control; DMSO: dimethyl sulphoxide; OD: optical density; SE: standard error).

Transmission electron microscopy (TEM) analysis was performed on GZNC sample to determine its features in nanometer domain as shown in Figure 1(g). It can be clearly seen that the graphene nanosheets were well decorated by ZnO nanoparticles, which densely and evenly deposited on both sides of these sheets to form a sandwich-like composite structure. Moreover, almost no ZnO nanoparticles were found outside of the graphene nanosheets. This indicates that the combination between ZnO nanoparticles and graphene nanosheets was almost perfect. The observed sheets were few layers and entangled with each other. Meanwhile, the Zn nanoparticles were identified in the range of ~9 nm and attached on both sides of graphene sheets with a nonuniform distribution on the sheet. The exposure of third instar larvae of transgenic* Drosophila melanogaster (hsp70-lacZ)Bg* ^*9*^ to 0.033 and 0.099 *μ*g/*μ*L of GZNC for 24 and 48 hr did not show any significant increase in the activity of *β*-galactosidase (Figure 2). However the larvae exposed to 0.199 and 3.996 *μ*g/*μ*L of GZNC showed a dose and time dependent significant increase in the expression of *β*-galactosidase (Figure 2).

**Figure 3:**
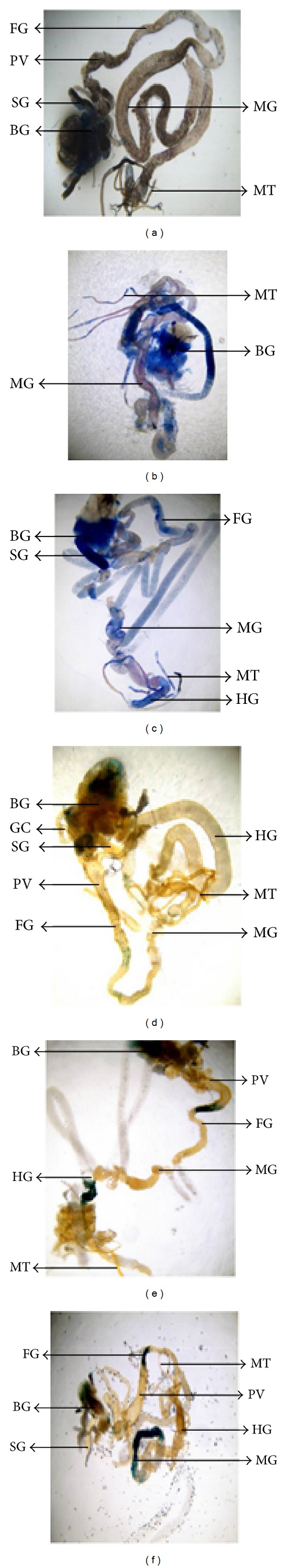
Trypan blue (a–c) and *β*-galactosidase (d–f) staining in the tissues of third instar larvae of transgenic* Drosophila melanogaster (hsp70-lacZ)Bg* ^*9*^ for untreated larvae (a, d) and the larvae exposed to different doses of graphene zinc nanocomposite (GZNC) for 48 hr of duration (0.199 *μ*g/*μ*L (b, e) and 3.996 *μ*g/*μ*L (c, f)) (BG: brain ganglia, SG: salivary gland, PV: proventriculus, FG: foregut, MG: midgut, HG: hindgut, MT: malpighian tubule, and GC: gastric caeca).

Figures 3(a), 3(b), and 3(c) show the trypan blue staining for the third instar larvae exposed to 0.199 and 3.996 *μ*g/*μ*L of GZNC for 48 hr of exposure. About 95% of untreated larvae were negative to trypan blue staining (Figure 3(a)). A dose dependent tissue damage was observed at both doses (i.e., 0.199 and 3.99 *μ*g/*μ*L) in brain ganglia, salivary glands, midgut, hindgut, and malpighian tubules. (Figures 3(b) and 3(c)). As compared to midgut the damage was more in the hindgut. The same results were obtained for 24 hr of exposure, but the staining intensity was less as compared to 48 hr of exposure (figures not shown). Figures 3(d), 3(e), and 3(f) show tissue with *β*-galactosidase staining in untreated larvae and the larvae exposed to 0.199 *μ*g/*μ*L and 3.991 *μ*g/*μ*L of GZNC for 48 hr. Dose dependent moderate to dark blue staining was observed for the exposure for 24 hr to 48 hr. The dark blue staining was prominent in the foregut and midgut region (Figures 3(e) and 3(f)).

**Figure 4:**
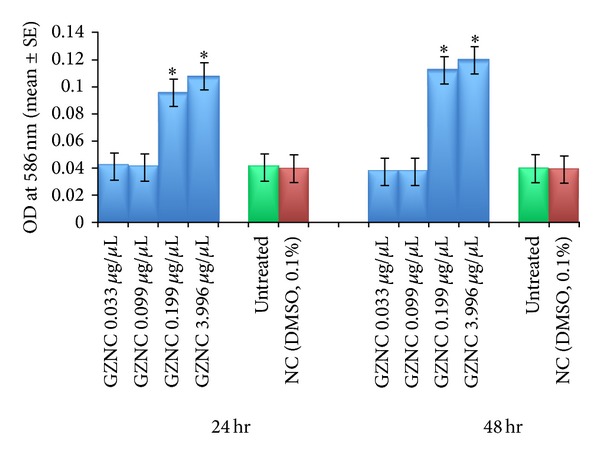
Lipid peroxidation in the third instar larvae of transgenic* Drosophila melanogaster (hsp70-lacZ)Bg* ^*9*^ exposed to different doses of graphene zinc nanocomposite (GZNC) for 24 and 48 hr. *Significant at *P* < 0.05 with respect to untreated larvae (GZNC: graphene zinc nanocomposite; NC: negative control; DMSO: dimethyl sulphoxide; OD: optical density; SE: standard error).

The results obtained for the estimation of lipid peroxidation are shown in Figure 4. No significant increase in the mean absorbance value for the exposure of third instar larvae to 0.033 and 0.099 *μ*g/*μ*L of GZNC was observed for 24 hr and 48 hr of exposure (Figure 4). The exposure of 0.199 and 3.996 *μ*g/*μ*L of GZNC for 24 hr was associated with the mean absorbance values of 0.096 ± 0.0005 and 0.108 ± 0.0029, respectively (Figure 4). The exposure of 0.199 and 3.996 *μ*g/*μ*L of GZNC for 48 hr was associated with the mean absorbance values of 0.113 ± 0.009 and 0.120 ± 0.0003, respectively (Figure 4).

**Figure 5:**
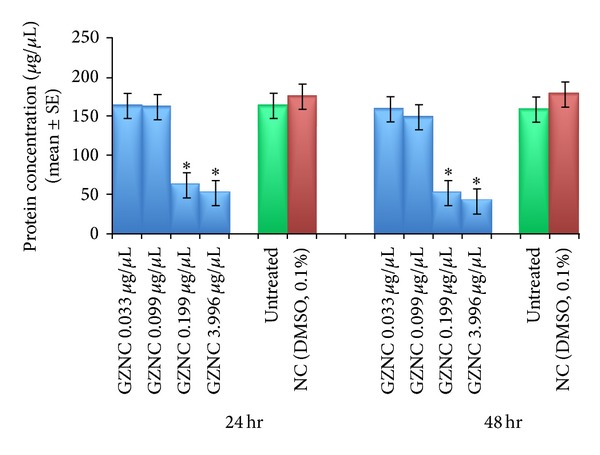
Protein content in the third instar larvae of transgenic* Drosophila melanogaster (hsp70-lacZ)Bg* ^*9*^ exposed to different doses of graphene zinc nanocomposite (GZNC) for 24 and 48 hr. *Significant at *P* < 0.05 with respect to untreated larvae (GZNC: graphene zinc nanocomposite; NC: negative control; DMSO: dimethyl sulphoxide; SE: standard error).

A significant decrease in the total protein content as compared to untreated larvae was observed in the larvae exposed to 0.199 and 3.996 *μ*g/*μ*L of GZNC for 24 hr and 48 hr of exposure (Figure 5). The exposure of 0.199 and 3.996 *μ*g/*μ*L of GZNC for 24 hr was associated with the total protein content (*μ*g/*μ*L) of 63.31 ± 0.270 and 52.61 ± 0.397, respectively (Figure 5).

**Figure 6:**
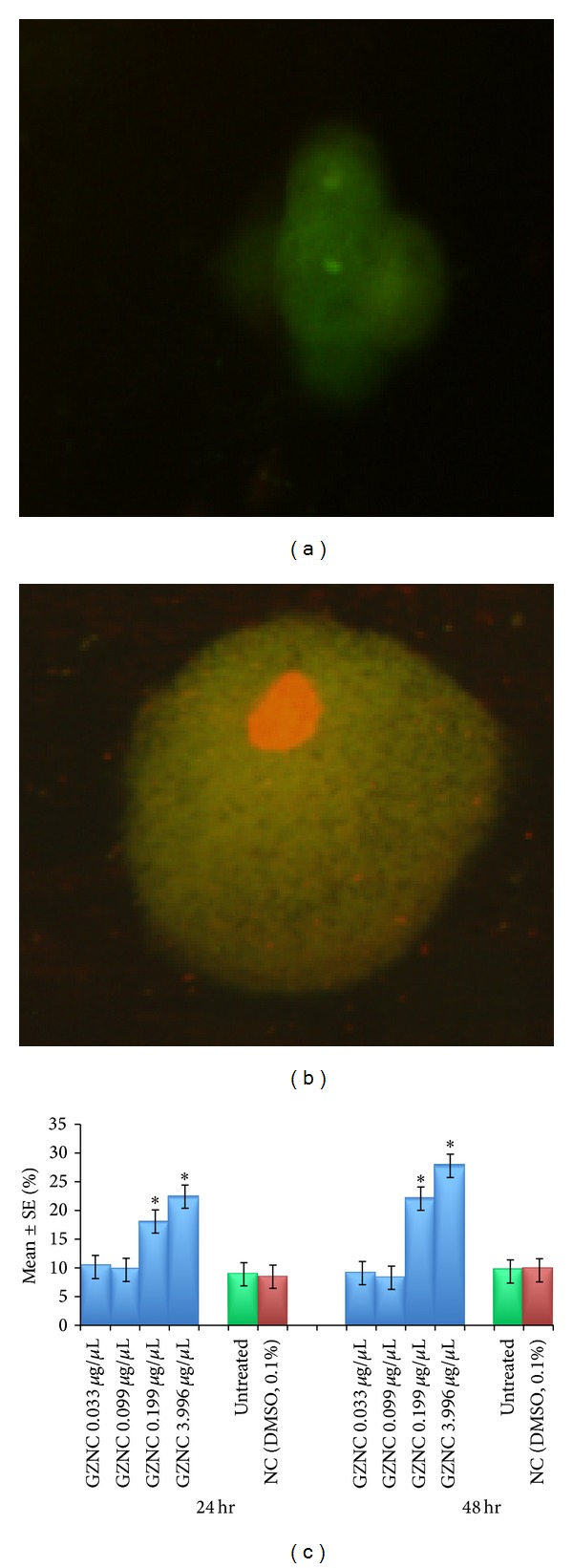
Midgut cells of third instar larvae of transgenic* Drosophila melanogaster (hsp70-lacZ)Bg* ^*9*^. (a) Normal cell; (b) apoptotic cell. (c) Apoptotic index measured in the midgut cells of the third instar larvae of transgenic* Drosophila melanogaster (hsp70-lacZ)Bg* ^*9*^ exposed to different doses of graphene zinc nanocomposite (GZNC) for 24 and 48 hr. *Significant at *P* < 0.05 with respect to untreated larvae (GZNC: graphene zinc nanocomposite; NC: negative control; DMSO: dimethyl sulphoxide; SE: standard error).

The normal and apoptotic midgut cells of the third larvae are shown in Figures 6(a) and 6(b). The exposure of 0.033 and 0.099 *μ*g/*μ*L of GZNC for 24 hr and 48 hr did not show any significant increase in the mean value for the apoptotic index as compared to untreated larvae (Figure 6(c)). The exposure of the third instar larvae for 24 hr and 48 hr at 0.199 and 3.996 *μ*g/*μ*L of GZNC showed a significant increase in the number of apoptotic cells in the midgut (Figure 6(c)). The exposure of 0.199 and 3.996 *μ*g/*μ*L of GZNC for 24 hr was associated with the mean value of 18.20 ± 0.860 and 22.60 ± 0.927 for apoptotic index, respectively (Figure 6(c)).

**Figure 7:**
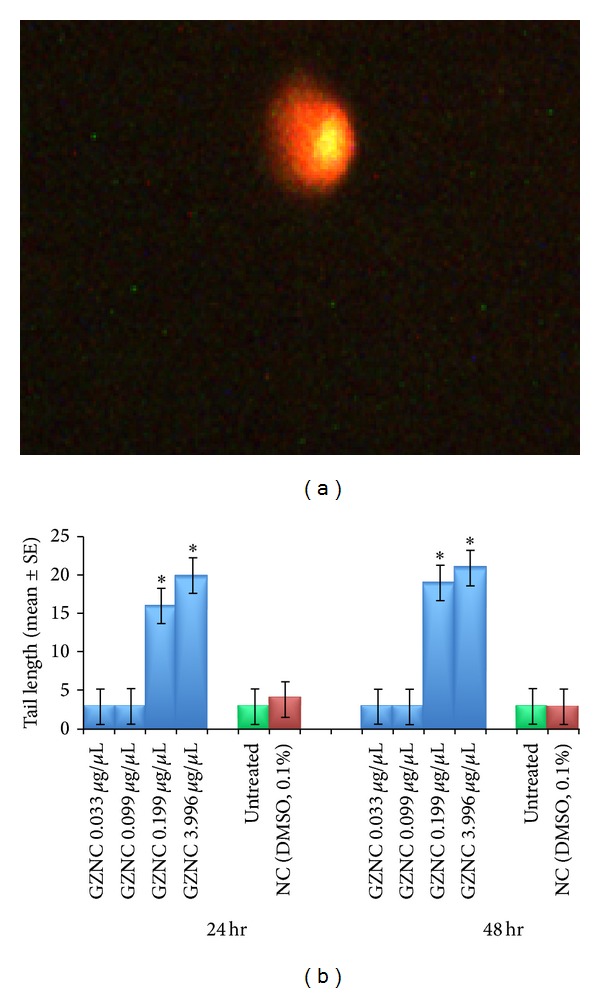
Comet assay performed on the midgut cells of the third instar larvae of transgenic* Drosophila melanogaster (hsp70-lacZ)Bg* ^*9*^ exposed to different doses of graphene zinc nanocomposite (GZNC) for 24 and 48 hr. *Significant at *P* < 0.05 with respect to untreated larvae (GZNC: graphene zinc nanocomposite; NC: negative control; DMSO: dimethyl sulphoxide; SE: standard error). (c) Comet assay performed in gut cell exposed to 3.996 *μ*g/*μ*L of GZNC for 48 hr of duration.

Comet assay performed on the midgut cell of the third instar larvae of transgenic* D. melanogaster (hsp70-lacZ)Bg* ^*9*^ is shown in Figure 7(a). The results obtained for the comet assay performed on the midgut cells of the larvae exposed to various doses of GZNC are shown in Figure 7(b). The exposure of 0.033 and 0.099 *μ*g/*μ*L of GZNC to the third instar larvae of transgenic* Drosophila melanogaster (hsp70-lacZ)Bg* ^*9*^ for 24 hr and 48 hr of duration did not show any significant increase in the mean tail length. The exposure of 0.199 *μ*g/*μ*L of GZNC for 24 hr and 48 hr was associated with the mean value of 16 ± 0.374 and 19 ± 0.374, respectively (Figure 7(b)). The exposure of 3.996 *μ*g/*μ*L of GZNC for 24 hr and 48 hr of duration was associated with the mean value of 20 ± 0.707 and 21 ± 0.678, respectively (Figure 7(b)).

## 4. Discussion

The results of the present study reveal that the doses of GZNC, that is, 0.199 and 3.996 *μ*g/*μ*L, are toxic to the third instar larvae of transgenic* Drosophila melanogaster (hsp70-lacZ)Bg* ^*9*^ for 24 hr and 48 hr of duration of exposure. Humans have exploited nanoparticles in small scale applications for centuries. Engineered nanoparticles have been found tremendously useful in a diverse array of industrial products, comprising personal hygiene, clothing, food industry products, paints, cosmetics, pharmaceuticals, electronics, and many more [22]. Pristine graphene was found to accumulate on the cell membrane causing high oxidative stress leading to apoptosis [23]. The interaction of nanoparticles with the biological systems is mostly unknown and this has led to the assessment of the toxicity of nanomaterials both* in vivo* and* in vitro* [24]. Graphene has attracted tremendous interest in different areas in recent years including biomedicine. The behaviour and toxicity of graphene have been extensively reviewed and the physicochemical properties such as surface functional groups, charges, coatings, sizes, and structural defects of graphene may affect* in vitro/in vivo* behaviour as well as its toxicity in biological systems [25]. A 10 *μ*g/kg body weight of graphene oxide was found to be toxic in mice after intravenous administration [26].

Zinc oxide nanoparticles have been reported to be cytotoxic even at very low concentration and exhibited strong protein adsorption abilities [27]. Cells of all organisms respond at the cellular level to all types of adverse changes in the environment such as temperature change, heavy metals, mutagens, and carcinogens by a protective mechanism called stress response or heat shock response [28]. Among the various families of stress gene,* hsp70* and its products are considered to be highly conserved and are extensively studied [29].

A hybrid gene that consists of the* Drosophila* heat shock gene,* hsp70*, fused to the* E. coli* beta-galactosidase gene has been introduced into the* Drosophila* germ line by the P-element microinjection method [10]. The *β*-galactosidase expression was found to be significantly higher at 0.199 and 3.996 *μ*g/*μ*L of GZNC for 24 hr and 48 hr of duration of exposure. Trypan blue staining showed the tissue damage at higher doses, that is, 0.199 and 3.996 *μ*g/*μ*L of GZNC for both 24 and 48 hr of duration of exposure. The concentration above 15 ppm of zinc oxide nanoparticles has been reported toxic to all human and rodent cells [30]. In earlier studies the exposure of zinc oxide nanoparticles to mice resulted in the damage of heart, lung, liver, and kidney [31]. A time and concentration dependent increase in the LPO product indicates the oxidative stress evoked by the 0.199 and 3.996 *μ*g/*μ*L of GZNC. LPO represents a reliable marker of free-radical generation and indicates the membrane damage [32]. Oxidative stress has been reported to damage membrane of lipid, protein, and DNA [33]. A significant time and concentration dependent decrease in the total protein content in the present study at 0.199 and 3.996 *μ*g/*μ*L of GZNC clearly demonstrates the damage of the protein, which can be correlated to the increase in LPO product. The present method used for the estimation of lipid peroxidation is based on the reaction of malondialdehyde (MDA) with 1-methyl-2-phenylindole at 45°C. Two molecules of 1-methyl-2-phenylindole react with one molecule of MDA to form a stable chromophore having maximal absorbance at 586 nm [34]. The major reactive aldehyde resulting from the peroxidation of biological membranes is malondialdehyde (MDA) [35].

A dose dependent decrease in the total protein content is clearly correlated with the increase in lipid peroxidation (*r* = −0.9975 (24 hr), *P* < 0.0007; *r* = −0.9976, *P* < 0.0007 (48 hr)) and apoptotic index (*r* = −0.9743 (24 hr), *P* < 0.0057; *r* = −0.98611, *P* < 0.0736 (48 hr)) (Table 1). Zinc oxide nanoparticles have been reported to induce cytotoxicity by increasing oxidative stress (increased levels of hydrogen peroxide and hydroxyl radicals and decreased levels of molecular oxygen and glutathione) in the human colon cancer cell lines [36]. The midgut cells were selected for the analysis of apoptosis and DNA damage by comet assay as these cells have been reported to be rich in cytochrome P-450 species and high microsomal oxidase activity [37]. A time and concentration dependent significant increase in the apoptotic cells and comet tail length at 0.199 and 3.996 *μ*g/*μ*L of GZNC clearly demonstrates the toxic effects of GZNC for 24 and 48 hr of exposure to the third instar larvae of transgenic* Drosophila melanogaster (hsp70-lacZ)Bg* ^*9*^. A positive correlation was observed in DNA damage and apoptotic index (*r* = 0.9935 (24 hr), *P* < 0.0086; *r* = 0.9861 (48 hr), *P* < 0.0736). A negative correlation observed between the *β*-galactosidase expression and protein content (*r* = −0.998 (24 hr), *P* < 0.0002; *r* = −0.9976 (48 hr), *P* < 0.003) clearly demonstrates the proteotoxicity in the larvae exposed to higher doses of GZNC (Table 1). In our earlier study with graphene copper nanocomposite in the third instar larvae of transgenic* Drosophila melanogaster (hsp70-lacZ)Bg* ^*9*^, GCNC showed toxic effects at 0.199 and 3.996 *μ*g/*μ*L for 24 hr of exposure and at 0.099, 0.199, and 3.996 *μ*g/*μ*L for 48 hr of exposure [17]. In our present study with GZNC the same doses were selected, but 0.099 *μ*g/*μ*L was found to be nontoxic for both 24 and 48 hr of exposure.

For the zinc oxide nanoparticles the main factors for the toxicity are sizes, surface, characteristics, dissolution, and exposure routes [3]. The use of nanomaterial into the matrices of polymer to make nanocomposites for wide variety of applications such as thin films for biosensors and biomedical devices [38], fibres for wound dressing [39], membrane for water purification [40], dispersing with antimicrobial properties [41], and many others is increasing in today's world due to low life cycle cost, design, flexibility, and applicability for a large scale fabrication [42]. It is the intrinsic property of the graphene such as tensile strength that has led it to be used in nanocomposites [6]. It has been reported that, due to its shape and structure, graphene and its oxides may show toxicity [2].

## 5. Conclusions

We have synthesized GZNC and a range of doses were studied on the third instar larvae of transgenic* Drosophila melanogaster (hsp70-lacZ)Bg* ^*9*^. It shows cytotoxicity as evidenced by (*hsp70* expression) as well as genotoxic damage (as evidenced by comet assay) in the midgut cells of the larvae. The toxicity of GZNC was observed only at 0.199 and 3.999 *μ*g/*μ*L for 24 and 48 hr of duration of exposure. Hence the full implementation of such nanomaterials in biological applications needs to be investigated more.

## Figures and Tables

**Table 1:** Regression analysis for *hsp70* induction, lipid peroxidation, protein level, apoptosis, and comet tail length in the third instar larvae of transgenic *Drosophila melanogaster (hsp70-lacZ)Bg^9^*.

S. number	Groups	24 hr				48 hr			
		Regression equation	*r*	*P*	*F*	Regression equation	*r*	*P*	*F*
1	*β*_gal_ versus L	*Y*_L_ = −0.0547 + 1.1112*X*_gal_	0.99965	<0.0007	2840.376	*Y*_L_ = −0.0739 + 1.2863*X*_gal_	0.99998	<0.000	47791.92
2	*β*_gal_ versus Ap	*Y*_Ap_ = −6.705 + 193.01*X*_gal_	0.98517	<0.0659	65.95374	*Y*_AP_ = −14.84 + 270.58X_gal_	0.98381	<0.0227	60.27102
3	*β*_gal_ versus CTL	*Y*_CTL_ = 14.202 − 11.96*X*_gal_	−0.5084	<0.2514	0.697138	*Y*_CTL_ = −21.28 + 278.95*X*_gal_	0.99991	<0.0000	10878.49
4	*β*_gal_ versus P	*Y*_P_ = 330.13 − 1927*X*_gal_	−0.9983	<0.0002	596.5014	*Y*_P_ = 307.64 − 1753*X*_gal_	−0.9976	<0.0003	415.9580
5	L versus Ap	*Y*_AP_ = 2.7587 + 174.09*X*_L_	0.98779	<0.3076	80.38286	*Y*_AP_ = 0.71960 + 210.10*X*_L_	0.98265	<0.9604	56.14397
6	L versus CTL	*Y*_CTL_ = −7.405 + 249.55*X*_L_	0.99883	<0.0035	851.3046	*Y*_CTL_ = −5.250 + 216.83*X*_L_	0.99980	<0.0015	4985.103
7	L versus P	*Y*_P_ = 235.19 − 1732*X*_L_	−0.9975	<0.0007	397.5076	*Y*_P_ = 206.95 − 1365*X*_L_	−0.9976	<0.0007	415.7933
8	Ap versus P	*Y*_P_ = 257.31 − 9.602*X*_AP_	−0.9743	<0.0057	37.48653	*Y*_P_ = 207.65 − 6.252*X*_AP_	−0.9786	<0.0064	45.20531
9	Ap versus CTL	*Y*_CTL_ = −10.98 + 1.4084*X*_AP_	0.99351	<0.0086	152.6177	*Y*_CTL_ = −5.454 + 1.000*X*_AP_	0.98611	<0.0736	70.47836
10	P versus CTL	*Y*_CTL_ = 26.344 − 0.1429*X*_P_	−0.9934	<0.0016	149.0998	*Y*_CTL_ = 27.605 − 0.1584*X*_P_	−0.9975	<0.0007	393.9279

*β*_gal_: *β*-galactosidase, L: lipid peroxidation, Ap: apoptosis, P: protein, and CTL: comet tail length.
